# Investigation on Compression Mechanical Properties of Rigid Polyurethane Foam Treated under Random Vibration Condition: An Experimental and Numerical Simulation Study

**DOI:** 10.3390/ma12203385

**Published:** 2019-10-17

**Authors:** Dacheng Qiu, Yannan He, Zhiqiang Yu

**Affiliations:** Department of Materials Science, Fudan University, 200433 Shanghai, China; 18210300053@fudan.edu.cn (D.Q.); 18110300027@fudan.edu.cn (Y.H.)

**Keywords:** rigid polyurethane foam, random vibration, compression performance, energy absorption properties, numerical simulation, modal analysis

## Abstract

The mechanical failure properties of rigid polyurethane foam treated under random vibration were studied experimentally and by numerical simulation. The random vibration treatments were carried out in the frequency range of 5–500 Hz, 500–1000 Hz, and 1000–1500 Hz, respectively. The influence of the vibration frequency, mass block and acceleration on the mechanical performance of rigid polyurethane foam was further investigated by compression testing. The experimental results showed that the compression performance and energy absorption of foams decreased the least between 500–1000 Hz. In addition, in the 5–500 Hz range, the reduction rate of compression performance and energy absorption increased with the increase of the vibration mass block and acceleration. The resulting simulation indicated that the deformation degree of the sample was the most serious under the condition of 5–500 Hz. With the increase of deformation, the damage of the sample during the vibration process increased, which led to the decrease of compression property and energy absorption of rigid polyurethane foam. This further explained the variation mechanism of the compression test performance.

## 1. Introduction

As a cellular material, polyurethane foam is often used in aerospace, petrochemical industry, packaging, and transportation due to its excellent mechanical properties and energy absorption properties [1]. Most applications of polyurethane foam are against compression loads [2,3]. Therefore, it is important to study the compression behavior of polyurethane foam. The mesoscopic structure of foams has an important effect on its compression behavior [2,4]. L. J. Gibson and M. F. Ashby had concluded that the stiffness of closed-cell foam material mainly comes from the edge of the cell, followed by the contribution of cell gas compression [2]. When compression loads were applied, the obturator foams would have the phenomena of bending of cell edges and folding of cell walls. In addition, foam density, strain rate, and ambient temperature also affect the compression performance of polyurethane foam [4,5,6]. Considering these factors, the researchers established a series of reliable constitutive models, such as an elastic-plastic constitutive model [5], elastic/crushable foam constitutive model [7], and hyperelastic/hyperfoam constitutive model [8]. Besides, based on these constitutive models, the compression behavior of polyurethane foam was predicted by using finite element analysis software. For example, Tae-Rim Kim et al. studied the compression behavior of polyurethane foam by using ABAQUS UMAT, which compiled an elastic-plastic constitutive model into a user-defined material subroutine [5]. 

At the same time, in the field of practical engineering, polyurethane foams are inevitably affected by light, temperature, humidity, load and other factors, leading to changes in its chemical composition and foam structure, and then the decline of its mechanical properties [9,10,11,12]. Thermal oxygen degradation caused by external factors is one of the most common reasons for polyurethane foam failure [10,11,13]. As the oxidation degradation progressed, the polyurethane molecular chain would hydrolyze, and the foam would change from toughness to brittleness, resulting in the foam bursting during the deformation process [11]. On the other hand, fatigue failure of polyurethane foam under external load (such as cyclic load) was one of the failure reasons, which was manifested by stiffness degradation and energy dissipation changes of foam materials [12,14,15]. The failure mechanism that the original crack induced abrupts brittle fracture under the action of load [16,17] and fracture toughness under different loads could be measured by the three-point bending test and shear test. 

The load form in the field of practical engineering is often complex and uncertain, among which the random vibration load is a typical load form. Random vibration refers to the vibration that cannot be described by deterministic function but has certain statistical rules [18]. The common random vibration in daily life is the vibration caused by the driving of a car. Soft polyurethane foam was often used as a cushion for automobiles [19,20,21], which could reduce and isolate the vibration of automobiles and make passengers more comfortable when driving. Flexible polyurethane foams have nonlinear and damping effects. Nonlinear stiffness and viscoelastic parameters of flexible polyurethane foams could be obtained by a vibration test and compression test [19,20]. However, beyond that, polyurethane foam also was often used as core material to make sandwich board [22,23] and honeycomb board structures [24], because of its good vibration absorption and isolation performance. To understand the vibration characteristics of foam materials, the researchers studied the density, strength, and viscoelasticity of foam materials [25,26]. Lyes Dib et al. found through the 3D COMSOL Multiphysics that Young’s modulus, Poisson’s ratio, damping factor, and density of polyurethane foam would change the absorption coefficient, thereby affecting the sound absorption performance [25]. The method of finite element numerical simulation could be used to analyze the material properties of foam materials under the vibration environment. 

At present, the researches on the vibration of polyurethane foam mainly focused on the nonlinear, damping effect, and vibration absorption and isolation of soft polyurethane foam. However, the study on vibration failure of polyurethane foam was not systematic and sufficient. In particular, the failure behavior of rigid polyurethane foam (RPUF) under random vibration and the numerical simulation of the random vibration process of the RPUF have not been reported yet. In this paper, the random vibration failure mechanism of the RPUF was adequately studied by means of experimental and finite element simulation. Random vibrations of different frequency ranges, mass block, and vibration acceleration were designed to simulate the actual working environment of the RPUF. The vibration failure degree of the RPUF was evaluated by the characterization of compression performance and energy absorption. The microstructure of the RPUF in the vibration direction was analyzed by field-emission scanning electron microscope (SEM). Moreover, the random vibration was explored and analyzed by using the ABAQUS 6.16 software step by step in the simulation process. The displacement curve and velocity curve at the top of the model under different frequency ranges were output by modal analysis, and the mechanism of vibration failure of the RPUF model was successfully explained by means of deformation and Von Mises stress distribution. 

## 2. Experimental

### 2.1. Materials and Vibration Processing

In this paper, the rigid polyurethane foams, with a density of 200 kg/m^3^, were obtained from polymethylene polyphenyl polyisocyanate (PAPI) with isocyanate value (NCO%) of 30.8% and polyether polyol with hydroxyl value (OH%) of 453.51mgKOH/g, and they were purchased from Shanghai Wantian Thermal Insulation Material Co., Ltd. The RPUF samples of 300 mm × 200 mm × 30 mm were subjected to random vibration processing, and the white noise spectrum was used to simulate the actual vibration environment. 

**Figure 1:**
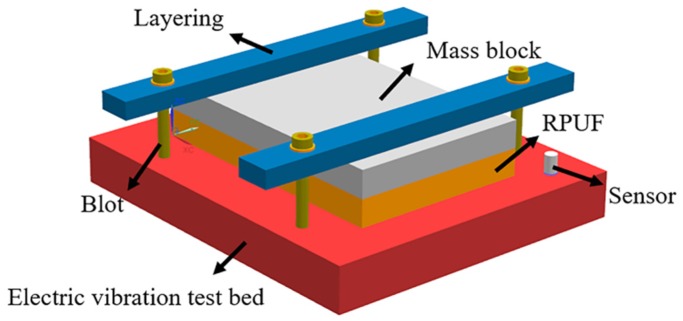
The random vibration testing system.

As shown in Figure 1, the random vibration testing system consisted of six parts: rigid polyurethane foam sample, mass block, layering, bolt, acceleration sensor, and high-frequency electric vibration testbed. Among them, the mass block was designed to simulate the bearing capacity of the foam material in actual working conditions. The purpose of the layering and bolting was to fix mass blocks and foam samples. The acceleration sensors monitored changes in acceleration during vibration. Finally, the high-frequency electric vibration testbed (ES-50LS4-445, Suzhou Dongling Vibration Test Instrument Co., Ltd., Suzhou, China) provided the vibration environment. 

The random vibration test is determined by four parameters: frequency range, power spectral density (PSD), total root-mean-square (RMS) acceleration, and test time. According to GB/T 4857.23-2012, the design of this experiment scheme is shown in Table 1. The random vibration test was conducted in the frequency range of 5–500 Hz, 500–1000 Hz, and 1000–1500 Hz, respectively. The effects of mass blocks and accelerations on rigid polyurethane foams were investigated in the range of 5–500 Hz. The alternating load of random vibration was applied in the z-axis direction for 1 h. 

### 2.2. Characterization and Testing

The micromorphology of the RPUF, after the random vibration, was observed by field emission scanning electron microscope (SEM, FE-SEM-4800-1, Japan Hitachi Technology Co., Ltd., Hitachi, Japan) in the direction of the vibration. The RPUF samples were cut into thin layers along the direction of vibration. Then, the thin layers were applied to the conductive adhesive and sprayed with gold for observation.

Furthermore, according to the GB/T 8813-2008, the quasi-static compression tests of RPUF after random vibration were carried out. The compression samples were 20 mm × 20 mm × 15 mm in size, and the compression tests were conducted according to the direction of vibration. Electronic universal testing machine (CMT4304-QY, MTS Systems Corporation, Minnesota, USA) was used for quasi-static compression tests with a strain rate of 0.001 s^−1^, and the tests ended at 80% strain. Each compression experiment was repeated five times; the average value as the experimental results. Next, the impact resistance and the energy absorption of RPUF were evaluated by energy absorption method [2,7]. According to the methods of U.E. Ozturk and G. Anlas [27], the absorbed energy-strain curve and energy absorption-stress curve were adopted to reflect the energy absorption performances of RPUF during the compression process. 

Absorbed energy (W) is defined as the energy absorbed by the foam material per unit volume. The unit volume work can be obtained by the area enclosed by the stress-strain curve.

(1) $$W=\int_{\varepsilon_{0}}^{\varepsilon_{f}}\sigma \left(\varepsilon_{f}\right)d\varepsilon_{f}$$

Energy absorption efficiency (P) is the ratio of the absorbed energy of the foam material to its corresponding stress.

(2) $$P=\frac{\int_{\varepsilon_{0}}^{\varepsilon_{f}}\sigma \left(\varepsilon_{f}\right)d\varepsilon_{f}}{\sigma \left(\varepsilon_{f}\right)}$$

where $\varepsilon_{0}$ is the starting strain and $\varepsilon_{f}$ is the terminal strain. In this paper, absorbed energy and the energy absorption efficiency were used to evaluate the energy absorption capacity of foam materials. These parameters took an average of five times the absorption of the compression energy. 

## 3. Numerical Simulation

**Figure 2:**
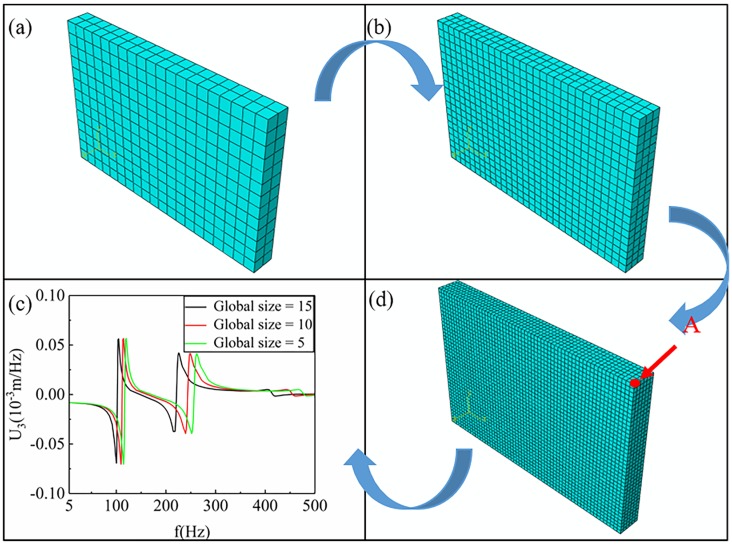
Convergence test and schematic diagram of the finite element model. (**a**) Global size = 15, (**b**) Global size = 10, (**c**) Convergence test, (**d**) Global size = 5.

The random vibration process was simulated by a commercial software ABAQUS 6.16. The convergence test was first carried out in this work to find out the most suitable mesh density, as shown in Figure 2. The vibration displacement curve of point A converges with the refinement of the mesh, and the curve obtained from the mesh of “global size = 10” is close to that obtained from the mesh of “global size = 5”. This means that the results obtained by using the mesh of “global size = 5” are convergent. Therefore, the mesh of “global size = 5” was adopted for the accuracy and computation efficiency in this work. In Figure 2d, the random vibration model was a rectangular specimen of 300 mm × 200 mm × 30 mm, and the mesh of the model consisted of C3D8R (An 8-node linear brick with a total of 14400 hexahedral units). In addition, point A is a vertex of the finite element model. The density of the RPUF was set at 2 × 10^−10^ t/mm^3^ (200 kg/m^3^), and its vibration direction was the z-axis. 

In this work, we adopted the method of E. Linul et al. [7], which used the elastic/crushable foam model as the constitutive relation model to characterize the overall mechanical properties of RPUF without random vibration treatment. The performance parameters of the RPUF samples without vibration treatment were obtained by the compression test. These parameters were assigned to the elastic/collapsible foam constitutive model to characterize the material properties of the RPUF. The Young’s modulus was set to 25.61 MPa and Poisson’s ratio to 0.2. The crushable foam model was used to describe the plastic state of materials with the volume-hardened yield surface. Yield surfaces evolved in a similar manner (constant α), and shape factor α was defined as

(3) $$\alpha =\frac{3k}{\sqrt{\left(3k_{t}+k\right)\left(3-k\right)}}$$

where k is the yield compressive stress ratio, and $k_{t}$ is the yield hydrostatic stress ratio. Therefore, the shape of the yield surface could be determined by defining k and $k_{t}$ values. The yield strength $\sigma_{b}$ was set to 1.67 MPa. 

In order to obtain the vibration characteristics of the samples, the modal analysis method was used to determine the modal and vibration modes of the rigid polyurethane foam samples. By ABAQUS/Standard, the Lanczos solver was used to calculate the natural frequency and the corresponding modes of the vibration model. The natural frequency eigenvalue of the undamped vibration model could be expressed as

(4) $$\left(-\omega^{2}M^{ab}+K^{ab}\right)\phi^{b}=0$$

where $M^{ab}$ for the quality matrix, $K^{ab}$ for the stiffness matrix, $\phi^{b}$ as the characteristic vector, a and b as the degrees of freedom. The Lanczos solver could be used to extract the first six natural frequencies and modes of the vibration model, with frequency ranges of 5–500 Hz, 500–1000 Hz, and 1000–1500 Hz, respectively. In this work, the acceleration base motion was employed as the boundary condition to constrain the freedom of the model. The degree of freedom was U_3_ (The direction of the vibration), and the amplitude was set as 0.129 g^2^/Hz, namely the acceleration power spectral density of random vibration. Furthermore, the stress of the sample during random vibration was simulated by means of the steady-state dynamics method, and the random vibration simulation of the RPUF further explained the variation of its compression test.

## 4. Results and Discussion

### 4.1. The Microstructure of Rigid Polyurethane Foam

**Figure 3:**
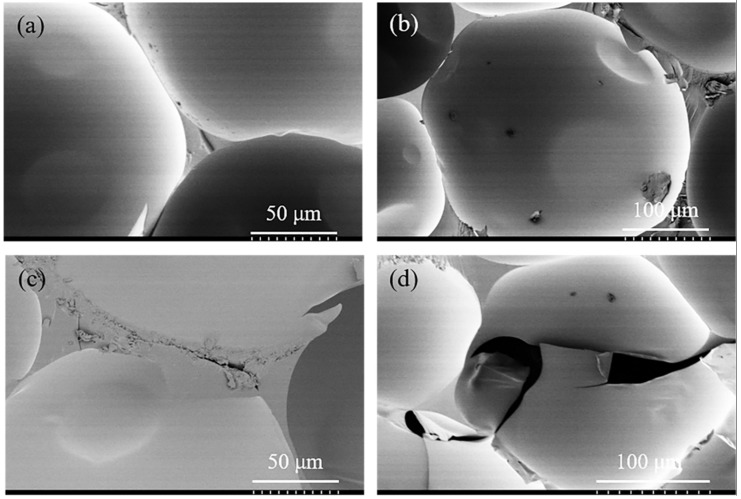
Microstructure (SEM) of the RPUF samples. Where, (**a**,**b**) is the sample without vibration, (**c**,**d**) is the sample after vibration.

The microstructure of rigid polyurethane foam without vibration treatment is shown in Figure 3a,b. It could be observed that the microstructure of the RPUF is mainly composed of triangular prismatic pillars and spherical foam cell bodies. Most of the cell bodies of the RPUF are spherical and densely distributed. There exist many small circular planes on the spherical cell body, which the "small window" formed by two cell bodies next to each other. The circular "small windows" are of different sizes, indicating different distances between the foam cells. As can be seen from Figure 3a,b, the triangular prismatic pillars of the foam are formed by three cell bodies adjacent to each other. These triangular prisms are the main load-bearing structures and provide the main stiffness of the foam material. 

In addition, the effect of vibration treatment on the rigid polyurethane foam was also observed, and the vibration damage mechanism was further analyzed. The micro-morphology of the sample after random vibration in the vibration direction is shown in Figure 3c,d. After random vibration, tiny cracks appeared on the prism of rigid polyurethane foam and its spherical cell bodies were broken. In Figure 3c, microcracks were first initiated at the pillar of the foam, and then propagated along the junction between the cells. This is mainly caused by the repeated compression of RPUF by mass block in the process of random vibration. As the main bearing structure, the cell edges underwent repeated bending until fatigue failure under the action of repeated compression, and then microcracks appeared at the edge of the cells. In Figure 3d, the foam cells burst laterally, and the cracked part of the foam wall curled and folded outwards to some extent. It is mainly ascribed to the pressure of the gas inside the closed-cell of foams. When the external vibration load reached the limit point, the pressure inside the cell of the foam materials was greater than the strength of the cell wall, which led to the tiny rupture of the cell body. Under continuous vibration, the cell body was repeatedly compressed by the mass block, which led to the microcrack growth on the cell body with the gas escaping from the cell body, and the cell body wall also crimped and folded. Hence the failure modes of rigid polyurethane foams under the random vibration environment mainly consist of two forms: microcrack of cell prism and cells burst.

### 4.2. Compression Properties of Rigid Polyurethane Foam

**Figure 4:**
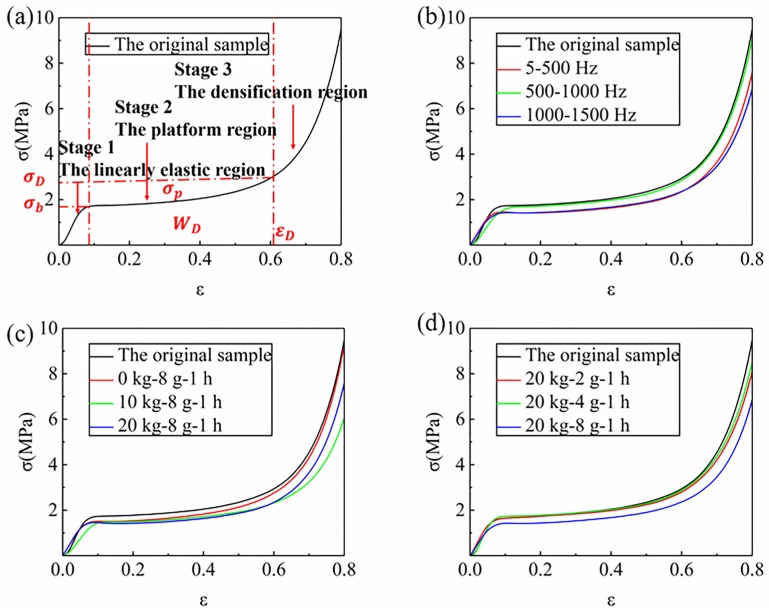
Compressive engineering stress-strain curves of the RPUF, (**a**) Schematic diagram of the foam compression stage, (**b**) Different frequency ranges, (**c**) Different mass blocks, (**d**) Different accelerations.

The compression stress-strain curves of the RPUF under different vibration conditions are shown in Figure 4. It can be seen from Figure 4a that the compression process of rigid polyurethane foam can be preliminarily divided into three stages: linear elastic stage, platform stage, and densification stage [7,28]. For the external impact and compression load, the performance of foam materials mainly depends on the energy absorption capacity of the platform stage [7]. Figure 4b shows the stress-strain curves of samples after the random vibration of three frequency ranges. Within 500–1000 Hz, the platform stress of the RPUF is significantly higher than that of the other two bands, which decreases by 6.0% compared with the original sample (The platform stress ($\sigma_{p}$) of the original sample is 2.01 MPa, from Figure 4a). However, the platform stress of the samples within 5–500 Hz and 1000–1500 Hz decreased by 17.4% and 13.4%, respectively. From Section 4.1, it could be seen that the failure forms of RPUF in the vibration environment mainly include cell edge cracking and cell bursting. The deformations are mainly achieved by the yield of cell prism and the compression of the cell body. With the compression load applies, the cell edges of the sample are gradually bent and the cells are gradually compressed. At this time, the microcracks in the cell edges gradually expand, and the cracked cells cannot bear the external load, which leads to a decrease of the stress of the platform. Therefore, this indicates that samples in the range of 5–500 Hz have the greatest damage in random vibration, while samples in the range of 500–1000 Hz have the least damage. 

Rigid polyurethane foams often suffer from random vibration of low frequency during practical application. Combined with the compared results of Figure 4b; therefore, the other influencing factors, mass block, and acceleration of the compression performance of the RPUF were further studied in the low-frequency range of 5–500 Hz. In Figure 4c, it could be seen that the $\sigma_{p}$ of the RPUF in the range of 5–500 Hz decreases with the increase of the quality of the mass blocks. When the mass block is not added, the $\sigma_{p}$ of the sample is 2.00 MPa, which decreases by 0.5% compared with the original sample. When the mass block of 10 kg is added, the $\sigma_{p}$ of the sample decreases by 9.5%, and the second increase of 10 kg is 17.4%. With the increase of mass block, the decreasing rate of platform stress increases gradually. In Figure 4d, the $\sigma_{p}$ of the samples with an acceleration of 2 g and 4 g decreases by 1.5% and 7.5%, respectively. In this case, the decreasing rate of the platform stress is low, and the damage degree of the sample is also minimal. The decrease of stress in the above platform is attributed to the decrease of energy absorption capacity of the materials caused by the microcracks in the foam prisms and the burst cells. 

**Figure 5:**
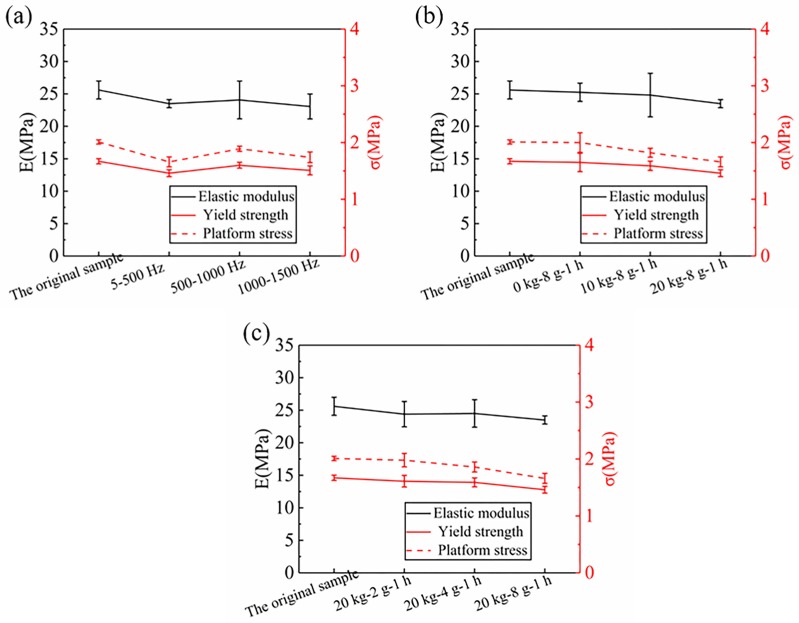
Compression properties of the RPUF under different vibration conditions, (**a**) Different frequency ranges, (**b**) Different mass blocks, (**c**) Different accelerations.

The damage of foam materials can be directly manifested by the change of its mechanical performance. Figure 5 shows the mechanical property parameters obtained by the stress-strain curves. Compression properties (elastic modulus (E), yield strength ($\sigma_{b}$)) of the RPUF all decrease to some extent under different vibration conditions. The E and $\sigma_{b}$ of the original sample are 25.61 MPa and 1.67 MPa, respectively. Compared with the original sample, the E and $\sigma_{b}$ of the samples in the 500–1000 Hz group decreased by 6.0% and 4.2%, respectively, whose rate of decline was the smallest of the three frequency ranges. Combined with the analysis in Figure 4, it is found that the platform stress, elastic modulus, and yield strength of the 500–1000 Hz samples all decreased. On the contrary, the E and $\sigma_{b}$ of samples with 5–500 Hz decreased by 8.2% and 12.6% compared with the original samples, respectively. The descending rate of the E, $\sigma_{b}$, and $\sigma_{p}$ of the 5–500 Hz samples is the largest in the three frequency ranges. 

In addition, it was also found that the decreasing rate of compression performance increases with the increase of the quality of the mass block. In the range of 5–500 Hz, the E and $\sigma_{b}$ of samples without mass block decrease by 1.4% and 1.2% compared to the original sample, respectively. When 10 kg mass block is added, the E and $\sigma_{b}$ of the sample decrease by 3.1% and 5.8%, then they decrease by 8.2% and 12.6% at 20 kg mass block, respectively. On the other hand, the vibration acceleration also affects the mechanical properties of the RPUF. When the acceleration is 2 g, the compression performance parameters of the samples decrease by 4.7% and 3.6% compared to the original sample, and by 4.3% and 4.8% when the acceleration is 4 g, respectively. 

**Figure 6:**
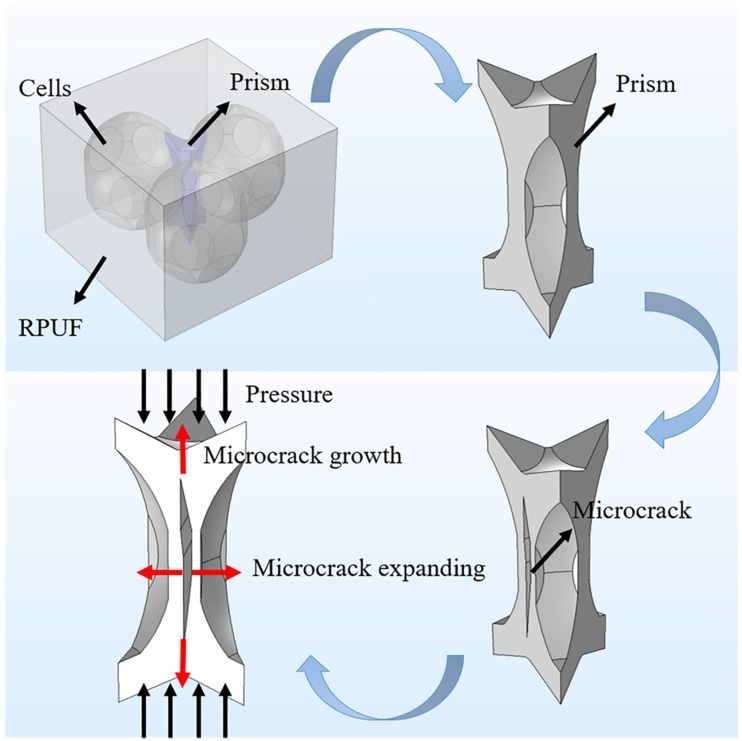
Schematic diagram of failure forms of cell prism (including microcracks) of rigid polyurethane foam.

The reason for the above difference in compression performance is the different damage degree of samples under different vibration conditions. The stiffness and strength of the RPUF are mainly provided by the cell prism during the linear elastic stage. When compression is applied, the cell prism gradually bends until a yield occurs. However, according to the analysis in Section 4.1, there are two defects in the foam after random vibration: the cracking of cell prism and the bursting of the cell. As shown in Figure 6, the prisms are formed by the proximity of three adjacent cells. With the compression of the foam material, the cell prism gradually bends, while the microcracks expand laterally and generate longitudinally. As a result, the cell prism could not effectively resist the external compression force, and the stiffness and strength of the RPUF decreased. In this section, the reduction in the compression performances of the 500–1000 Hz is the smallest, while that of 5–500 Hz is the largest. In the range of 5–500 Hz, the compression performances of the RPUF decrease with the increase of quality of mass block and vibration acceleration. This indicates that the damage degree of samples in the range of 500–1000 Hz is the least, while the damage degree of thd samples in the range of 5–500 Hz is the most serious. Moreover, the increase of mass block and acceleration aggravates the damage degree of the sample. 

### 4.3. Energy Absorption of Rigid Polyurethane Foam

Vibration and collision often occur in the field of packaging and transportation. At this point, energy is absorbed mainly through the cushioning deformation of polyurethane foam. The deformation is mainly achieved by the yield of the cell prism and the compression of the cell body, and the damage of the foam cell body has a great influence on the deformation. Therefore, it is of great practical significance to evaluate the energy absorption capacity of rigid polyurethane foam. By using the methods presented by U. E. Ozturk and G. Anlas [27], the absorbed energy-strain diagram and energy absorption efficiency-stress diagram of the RPUF treated under different vibration conditions were obtained. These two curves are used to reflect the energy absorption of foam materials during compression. 

As shown in Figure 5, where Figure 5a,b show the absorbed energy-strain curve and energy absorption efficiency-stress curve within three frequency ranges, Figure 5c,d are those of the foam samples with different mass blocks within the frequency range of 5–500 Hz, and Figure 5e,f reveal the energy absorption of samples with different vibration accelerations at the frequency range of 5–500 Hz, respectively. It could be seen that the absorption energy of the samples without vibration increases steadily as the strain increases when the strain is below 0.6, but it increases rapidly when the strain is above 0.6 and below 0.7. It resulted in the transition from the platform stage to the densification stage between the strain of 0.6 and 0.7, which led to a sharp increase in the amount of absorbed energy. On the other hand, the energy absorption efficiency of the sample that didn’t vibrate increased sharply around 2 MPa, and when it reached a maximum, the energy absorption efficiency decreased gradually. This is because that the RPUF yields gradually with the increase of compression load, and its stress-strain curve has the stage of platform zone. The stress value in the platform area basically remains unchanged with the increase of strain. At this time, more energy is absorbed by unit stress. After reaching the densification stage, the stress value increases sharply with the increase of strain. However, the energy absorbed per unit of stress decreases. Therefore, there exists a maximum energy absorption efficiency during the process of transition from the platform stage to the densification stage, and the corresponding stress value is the starting point of the densification stage. 

**Figure 7:**
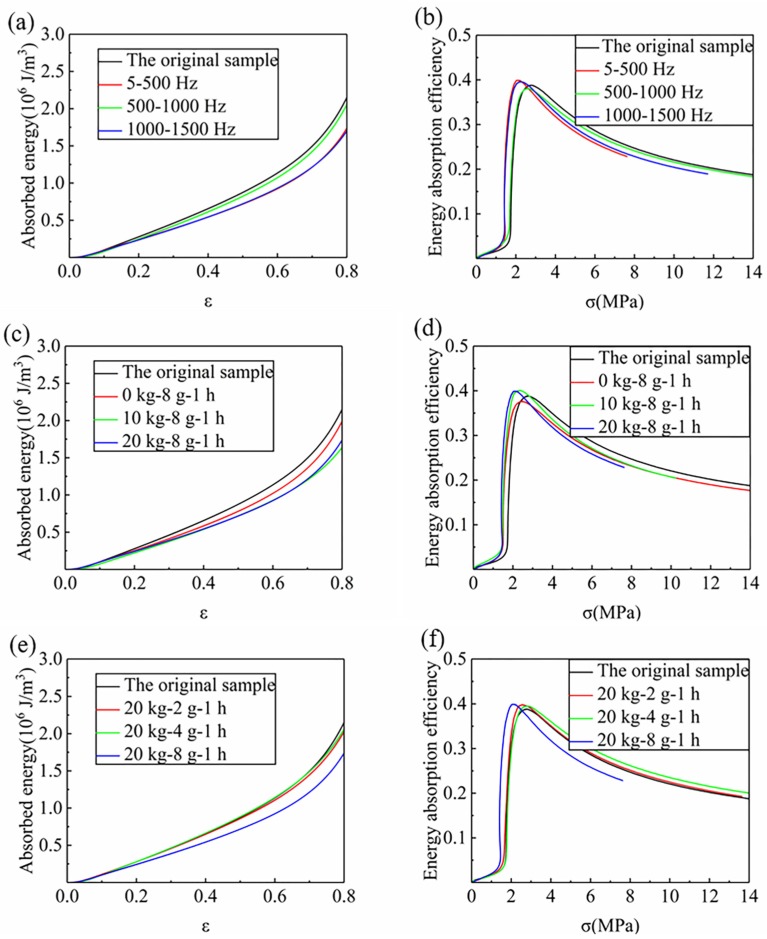
Absorbed energy-strain curve (**a**,**c**,**e**) and energy absorption efficiency-stress curve (**b**,**d**,**f**) of the RPUF under different vibration conditions, (**a**,**b**) Different frequency ranges, (**c**,**d**) Different mass blocks, (**e**,**f**) Different accelerations.

In comparison, it could be found from Figure 7a that the slope of the absorbed energy-strain curves of the samples in the three frequency ranges are all smaller than that of the original samples, but the slope of the sample in the 500–1000 Hz range is higher than that of the samples in the other two frequency ranges. It suggests that the energy absorption properties of the RPUF all decrease at three different vibration frequency ranges and the decrease of samples in the range of 500–1000 Hz was relatively smaller. The absorbed energy-strain curves of different mass blocks and vibration acceleration present a similar variation trend compared with those of different vibration frequencies, respectively. It could be seen from Figure 7c that the slope of the absorbed energy-strain curve of the sample decreases with the increase of the mass block in the range of 5–500 Hz but the slope of a mass block of 10 kg is basically the same as that of the 20 kg, showing that the effect of the low mass of a block on the energy absorption property of the RPUF is obvious. In Figure 7e, the slopes of the absorbed energy-strain curves of the samples with accelerations of 2 g and 4 g are all close to that of the samples without vibration. However, when the acceleration is 8 g, the slope of the absorbed energy-strain curve presents a significant decrease compared to the sample without vibration, indicating that only higher vibration acceleration has an obvious influence on the energy absorption performance. 

The energy absorption of the RPUF during the compression process is mainly provided by the platform stage. Combined with Section 4.2, it can be found that the variation trend of the slope of the absorbed energy-strain curve is similar to the platform stress. The higher the stress of the platform, the more energy the foam absorbs, and the higher the slope of the absorbed energy-strain curve. It is mainly ascribed to the degree of damage caused by different vibration conditions are not the same. The burst cells are unable to absorb external energy, and the samples rapidly change from the platform stage to the densification stage, which led to the decrease of the platform stress and slope of the absorbed energy-strain curve. 

**Figure 8:**
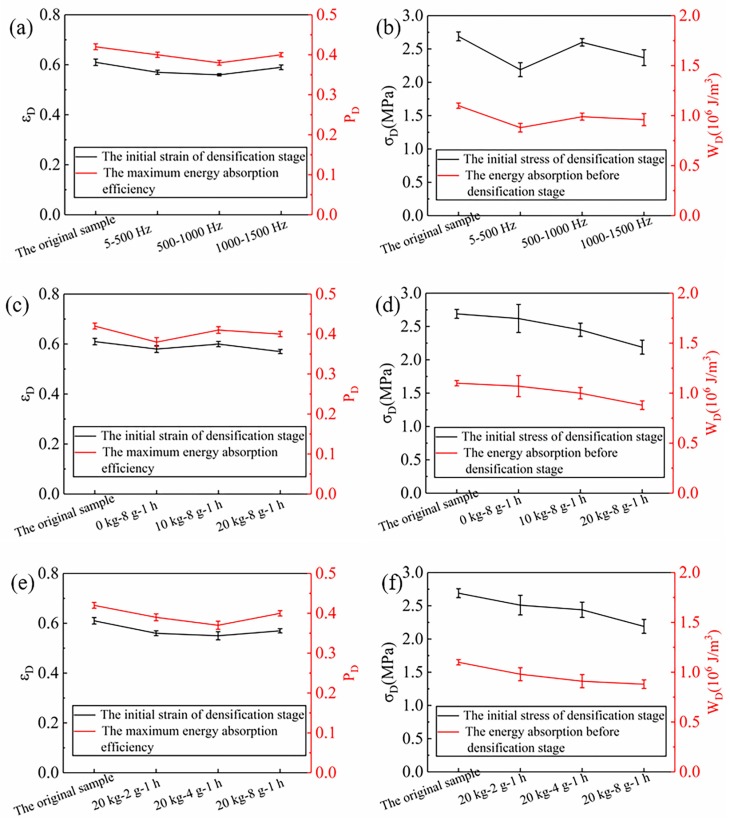
Compression energy absorption properties of the RPUF under different vibration conditions, (**a**,**b**) Different frequency ranges, (**c**,**d**) Different mass blocks, (**e**,**f**) Different accelerations.

The stress value corresponding to the maximum energy absorption efficiency ($P_{m}$) in Figure 7b,d,f is defined as the initial stress ($\sigma_{D}$) in the densification stage. Therefore, the initial strain ($\varepsilon_{D}$) of the densification stage could be determined by Figure 4, and the total absorbed energy ($W_{D}$) before the densification stage can be obtained by the absorbed energy-strain curve in Figure 7a,c,e. In Figure 8, the energy absorption performance parameters obtained by statistics are shown. It can be seen clearly that under different vibration conditions, the $\varepsilon_{D}$ in the densification stage is all slightly smaller than that of the original sample, which indicates that the densification stage of the sample treated under vibration appears slightly earlier. Similarly, compared with the non-vibrating samples, the maximum energy absorption efficiency ($P_{D}$) of the samples under different vibration conditions also decreases slightly. It is because the initial strain and the maximum energy absorption efficiency of the densification stage are changed due to the damage in the vibration process. 

In the three different frequency ranges, the initial stress ($\sigma_{D}$) of the densification stage and $W_{D}$ of the samples in the 500–1000 Hz group at the initial point of the densification stage increases all slightly. They decreased by 3.3% and 10.0%, respectively, compared tothe samples without vibration. It is ascribed to the change of the platform stress, which leads to a decrease of the stress and absorbed energy at the initial point of the densification stage. In addition, it also can be found that within the frequency range of 5–500 Hz, the $\sigma_{D}$ and $W_{D}$ are all decreases with the increase of quality of mass blocks and vibration acceleration. When the mass block is 10 kg, these two performance parameters are 2.45 MPa and 1.00 × 10^6^ J/m^3^, decrease by 6.4%, and 6.5% compared to no mass block (0 kg) at an acceleration of 8 g and 1 h vibration time, respectively. Under the condition of the same mass (20 kg) of the vibration block and vibration time (1 h), the two parameters of the sample with an acceleration of 4 g are reduced by 2.8% and 7.1%, respectively, compared with those of 2 g, respectively. 

During random vibration, rigid polyurethane foam is subjected to repeated compression by mass blocks. As the quality of the mass block increases, the inertia of mass blocks is gradually strengthened, which aggravates the damage of rigid polyurethane foam. With respect to the effect of the change in acceleration is due to an increase in acceleration that strengthens the pressure-compression effect of the mass block on the rigid polyurethane foam. In this way, the damage of the RPUF is aggravated. The cells in the foam at the platform stage bend and yield and the microcracks grow, which leads to the bending and breaking of the prism. On the one hand, this leads to the advance of the densification stage, and the stress required to reach the densification stage is reduced. The broken prisms, on the other hand, stand in a staggered way, which leads to the increase of the number of prisms under load, on the contrary, thus increasing the stiffness of the RPUF and slowing down the decrease of energy absorption to some extent. 

### 4.4. Finite Element Simulation of the Vibration Process of Rigid Polyurethane Foam

The random vibration process of the RPUF in three different frequency ranges is simulated by using a commercial software ABAQUS 6.16. By means of modal analysis, the vibration characteristics of the RPUF in three frequency ranges were analyzed, and the modes were obtained. It was found that the vertex of the finite element model had the largest displacement along the vibration direction in each mode. One of the vertices (defined as point A, see Figure 2) of the finite element model was selected for research in this paper. By analyzing the displacement and velocity at point A, the deformation of the finite element model in three frequency ranges could be obtained, which lays the foundation for the subsequent steady-state dynamic analysis. The deformation degree of the finite element model reflects the damage of the sample in the random vibration process to some extent. When the deformation of the model is serious, the damage of the sample is also serious, which leads to the significant decline of its compression and energy absorption properties. 

**Figure 9:**
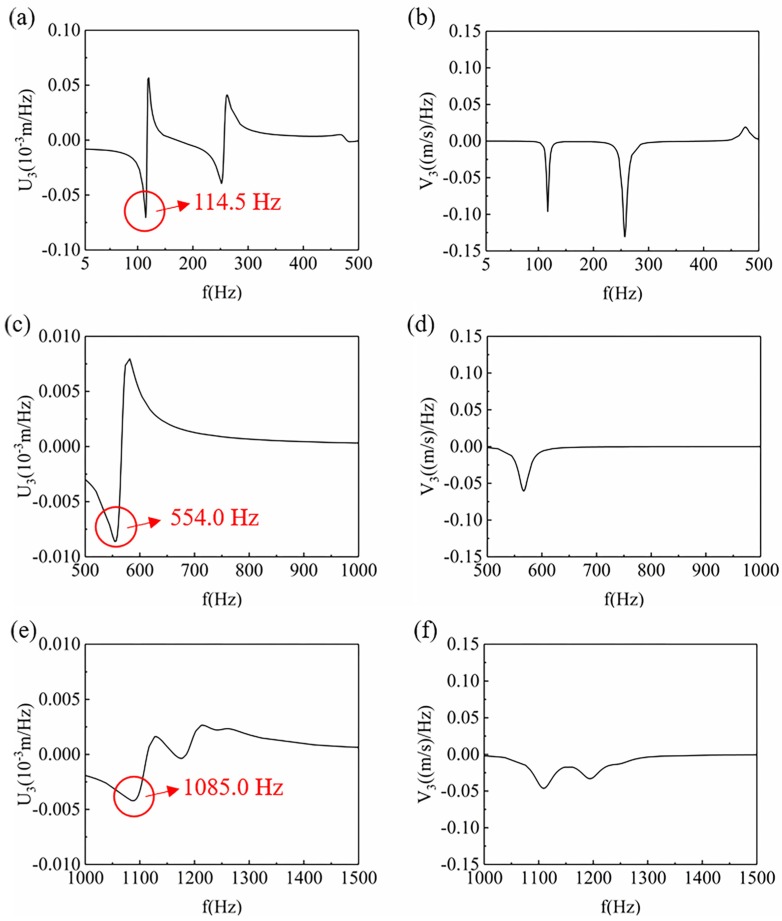
The displacement (**a**,**c**,**e**) and velocity (**b**,**d**,**f**) curves of the z-axis at point A of the finite element model within three frequency ranges, (**a**,**b**) 5–500 Hz, (**c**,**d**) 500–1000 Hz, (**e**,**f**) 1000–1500 Hz.

In this paper, the curves of displacement (Figure 9a,c,e) and velocity (Figure 9b,d,f) of point A were used to characterize the deformation of the model. In Figure 9, the statistical relationship between the displacement and frequency and between the velocity and frequency is characterized by displacement and velocity power spectral density, respectively. Based on the analysis of the displacement power spectral density of point A, the deformation of the finite element model in three frequency ranges. Figure 9a,c,e, which shows the displacement of the power spectral density of point A can be obtained in the frequency range of 5–500 Hz, 500–1000 Hz, and 1000–1500 Hz, respectively. 

In comparison, the maximum value of response displacement at point A in the frequency range of 5–500 Hz is larger than those of the other two frequency ranges, followed by the range of 500–1000 Hz. Besides, the maximum value of response displacement at point A in the range of 1000–1500 Hz is the smallest. It can be preliminarily inferred that the deformation of the RPUF samples is the largest in the range of 5–500 Hz, and the smallest in the range of 1000–1500 Hz. In addition, it also can be seen that there are double peaks and troughs in the range of 5–500 Hz and 1000–1500 Hz, and only one pair of peaks and troughs in the range of 500–1000 Hz. This indicates that in the range of 5–500 Hz and 1000–1500 Hz, the response displacement of random vibration changes more frequently; that is, the deformation times of the finite element model are more frequent in these two frequency ranges. However, the response displacement changes less in the range of 500–1000 Hz, so the deformation times are less. With the deepening of deformation degree and the increasing of deformation times, cell edge cracking and cell bursting are more likely to occur in the vibration process due to the pressure of mass blocks. Therefore, the damage of the model is the most serious in the range of 5–500 Hz, while the damage of the model is the least in the range of 500–1000 Hz. Besides, in the range of 1000–1500 Hz, the deformation of the model is minimal, but there are many times of deformation, which deepens the damage of the model. 

Figure 9b,d,f shows the velocity power spectral density of point A in the frequency range of 5–500 Hz, 500–1000 Hz, and 1000–1500 Hz, respectively. By analyzing the maximum response speed of the model, and the width of the trough, the deformation speed of the model in three frequency ranges can be inferred. In the range of 5–500 Hz, the model has one crest and two troughs, among which the troughs are the sharpest. This indicates that the model has two small and rapid deformation frequencies in the range of 5–500 Hz, and the damage to the RPUF is relatively large. However, there are two broad troughs in the range of 1000–1500 Hz. It suggests that the two deformation frequency regions of the model in this range are wide; that is, the deformation time is long, which deepens the damage of the model. The model has only one trough in the range of 500–1000 Hz. Through the above analysis, it can also be concluded that the damage of the models in the range of 5–500 Hz and 1000–1500 Hz is relatively serious, while the damage of the models in the range of 500–1000 Hz is relatively mild. 

**Figure 10:**
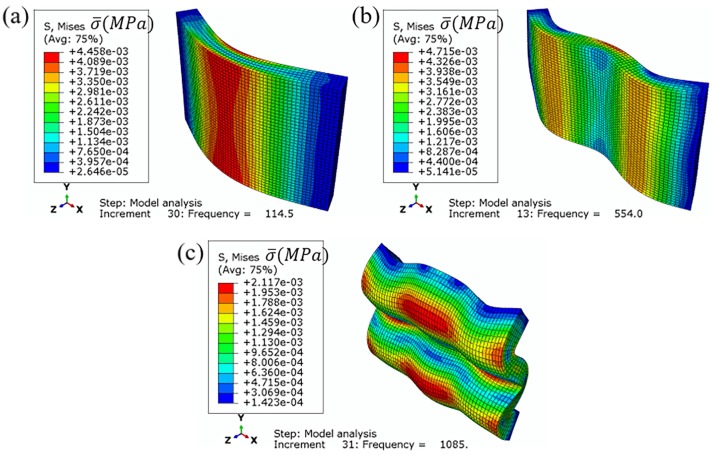
Mises stress cloud of finite element model, (**a**) 114.5 Hz, (**b**) 554.0 Hz, (**c**) 1085.0 Hz.

Besides, in Figure 9, the frequencies of the maximum displacement point in the three frequency ranges were 114.5 Hz, 554.0 Hz, and 1085.0 Hz, respectively. In order to realize the stress distribution of the model at the maximum deformation, the steady-state dynamic analysis of the finite element model was carried out. Three frequency points, 114.5 Hz, 554.0 Hz, and 1085.0 Hz, were selected for the simulation analysis of the random vibration process. In Figure 10, with the increase of frequency, the deformation form of the model changes from a single direction of bending to a combination of three directions. The model is bent once in the x-axis direction at 114.5 Hz. At 554.0 Hz, the model is bent twice in the x-axis direction, and at 1085.0 Hz, the model is bent in a complex way. The more complex the deformation is, the more deformation parts exist in the foam. This means that the wider the deformation range is, the more likely the specimen is to be destroyed. On the other hand, combined with Figure 9, the sample with a simple deformation form is more likely to produce larger response displacement. 

Besides, it can also be seen from Figure 10 that the maximum values of Mises stress are mainly located at the bending deformation of the models. The distribution of Mises stress is related to the deformation of the model. Taking a model at 114.5 Hz, as an example, the Von Mises stress starts from the bending position and decreases along both sides. At 114.5 Hz and 554.0 Hz, the model deformation mode is relatively simple with fewer bending positions, resulting in a wide range of Von Mises stress distribution and a large number of low-stress areas. However, at 1085.0 Hz, the model deformation is of a complex form with a large number of bending parts, resulting in that most of the Von Mises stress is around the position of bending deformation and few low-stress areas. Therefore, this means that the distribution of the Von Mises stress at 1085.0 Hz is relatively centered, and the damage of the random vibration to rigid polyurethane foam is relatively large. Among them, the model stress values at 114.5 Hz and 554.0 Hz are generally much larger than those at 1085.0 Hz, which the reason for the large deformation of rigid polyurethane foam at these two frequencies. 

To sum up, the deformation caused by random vibration is the largest within the range of 5–500 Hz, the stress concentration is also the largest, and the structural damage of RPUF is the most serious. However, the number of deformation changes in the model in the range of 500–1000 Hz is the smallest, and the range of stress concentration is also the smallest. Hence the RPUF damage in 500–1000 Hz is the least. They are consistent with the compression testing results. This indicates that the deformation and stress concentration of the RPUF in the vibration process are the main causes of foam failure.

## 5. Conclusions

In this paper, the compression properties of rigid polyurethane foams under random vibration were studied, and the random vibration processes of foams in different frequency ranges were simulated by ABAQUS 6.16. The results showed that the microcracks initiation of cell prisms and the rupture of cell bodies occurred in the RPUF during random vibration. As a result, its compression performance and energy absorption decreased to varying degrees. In the frequency range of 5–500 Hz, the compression property and energy absorption of the RPUF were the most severe declines, and those of the RPUF decreased the least in the range of 500–1000 Hz. In the range of 5–500 Hz, the increase of the quality of the mass block and vibration acceleration would deepen the damage of the RPUF, resulting in the decline of its compression performance and energy absorption. In the numerical simulation, the deformation of the RPUF was the most serious in the range of 5–500 Hz. This further indicated that, after treatment under random vibration, the samples of 5–500 Hz suffered the most serious damage in three frequency ranges. On the whole, the results obtained by the experiment and the simulation were consistent with each other, thus proving that the compression performance and energy absorption of RPUF would decrease due to the damage caused by random vibration.

## Figures and Tables

**Table 1:** Conditions for random vibration tests.

Number	Frequency Range (Hz)	Mass Block (kg)	RMS Acceleration (g/Hz)
1	5–500	20	8
2	500–1000	20	8
3	1000–1500	20	8
4	5–500	0	8
5	5–500	10	8
6	5–500	20	2
7	5–500	20	4
